# Management of Upper Airway Infantile Hemangiomas: Experience of One Italian Multidisciplinary Center

**DOI:** 10.3389/fped.2021.717232

**Published:** 2021-12-07

**Authors:** Marialuisa Corbeddu, Duino Meucci, Andrea Diociaiuti, Simona Giancristoforo, Roberta Rotunno, Michaela Veronika Gonfiantini, Marilena Trozzi, Sergio Bottero, May El Hachem

**Affiliations:** ^1^Dermatology Unit and Genodermatosis Unit, Genetics and Rare Diseases Research Division, Bambino Gesù Children's Hospital, IRCCS, Rome, Italy; ^2^Airway Surgery Unit, Bambino Gesù Children's Hospital, IRCCS, Rome, Italy; ^3^Rare Diseases and Genetic Unit, Bambino Gesù Children's Hospital, IRCCS, Rome, Italy

**Keywords:** airway, infantile hemangioma (IH), endoscopy, stridor, propranolol

## Abstract

Airway infantile hemangiomas (IHs) can represent a life-threatening condition since the first months of life. They may be isolated or associated to cutaneous IHs, and/or part of PHACES syndrome. Diagnosis, staging, and indication to treatment are not standardized yet despite the presence in the literature of previous case series and reviews. The diagnosis might be misleading, especially in the absence of cutaneous lesions. Airway endoscopy is the gold standard both for diagnosis and follow-up since it allows evaluation of precise localization and entity of obstruction and/or stricture. Proliferation of IH in the infant airways manifests frequently with stridor and treatment is required as soon as possible to prevent further complications. The first line of therapy is oral propranolol, but duration of treatment is not yet well-defined. All considered, we report the experience of our multidisciplinary center from 2009 to date, on 36 patients affected by airway IHs, and successfully treated with oral propranolol. Thus, the authors propose their experience for the management of airway IHs, specifically early diagnosis, when to perform endoscopy, how to interpret its findings, and when to stop the treatment.

## Introduction

Infantile hemangiomas (IHs) are the most common benign vascular tumors in children and are a neoplastic proliferation of endothelial cells. They are characterized by a growth phase that consists in a rapid proliferation of blood vessels in the first months of life. Afterward, a spontaneous gradual involution, partial or complete, with or without scarring and in some cases with a residual fibrofatty tissue, usually continues until 5–7 years of age. They are benign and self-limited, but sometimes they can manifest complications such as ulceration, permanent disfigurement, and they may compromise vital organs causing functional and life-threatening damages (1). Most IHs are not clinically evident at birth and sometimes they may be preceded by a cutaneous mark, more frequently an area of pallor at the hemangioma site. Airway IHs (AIHs) constitute a rare occurrence with an unknown incidence. They may be associated with cutaneous IH with a particular distribution in 50% of cases, called “beard” area or S3 area, according to Haggstrom and Frieden's classification (2). This distribution includes the preauricular skin, mandible, lower lip, chin, and/or anterior neck. However, they can also develop in children in the absence of cutaneous hemangiomas. Moreover, recent studies demonstrated that AIHs might be associated with more limited skin patterns, as well as with posterior and lateral neck involvement (3).

PHACES syndrome is characterized by posterior fossa anomalies, IH, aortic, cardiac, and eye malformations, and sternal defect. Several studies reported its association with AIHs with variable percentages (16–50%) (4–6). AIHs usually involve the subglottis, which is the narrowest part of the pediatric airway and are mostly located in its left posterolateral part (7, 8). The infants manifest frequently progressive hoarseness or biphasic stridor, especially between the age of 6 and 12 weeks, with a possible evolution to respiratory failure. Respiratory distress is caused by external compression or IH localization within an airway tract that could cause a partial or total obstruction. Other symptoms include cough, sleep apnea, and failure to thrive. Symptoms are often mistaken as infectious or inflammatory croup, bronchiolitis, laryngitis, allergy, tracheomalacia, or tumor (9).

Differential diagnosis is based on the peculiar characteristics of stridor associated with AIHs, which is usually biphasic and worsen despite non-specific treatments. Furthermore, time of onset of respiratory symptoms is typical and differs from other congenital airway diseases, in fact it is generally absent at birth and usually develops at 2–4 months of age (10). In a child with noisy breathing, stridor, or respiratory distress a complete endoscopic evaluation is crucial and early intervention is mandatory to prevent life-threatening complications.

The first step is to perform an awake trans-nasal fiber-optic laryngoscopy to evaluate laryngeal motility and pharyngo-laryngeal functional aspects. The assessment of the upper and lower airways is completed with an asleep endoscopy, performed under anesthesia while maintaining spontaneous breathing, using sevoflurane or intravenous propofol. The occurrence of laryngospasm passing the glottis with the endoscope, could be prevented by using laryngeal local anesthesia. Usually, in our experience, a 3.5-mm video-bronchoscope, passed through the nose, is used in infants, while a 2.2-mm slim fiber-optic bronchoscope in newborns. In case of suspected subglottic or tracheal stenosis, a direct laryngo-tracheoscopy by rigid endoscope is indicated to obtain a clear view of the subglottis and the trachea. Endoscopy is usually performed using a 4 mm-diameter rigid fiber-optic or 2 mm, based on patient age or size of the stenotic tract. This procedure grants the identification of the AIH, its location, extent, and degree of obstruction. The endoscopic features of AIH are very characteristic. It usually appears as a red-violet mass, with a smooth and regular surface, easily compressible. When the IH involves the subglottic space, measurement of the degree of stenosis, and percentage of obstruction is achieved passing endotracheal tubes of different sizes through the stricture, then comparing results with the expected airway size for age (11).

Therapeutic options before the introduction of oral propranolol included tracheotomy, CO_2_ laser, systemic or intralesional corticosteroids, and/or open surgical excision. Oral propranolol is now considered the mainstay of treatment, since it is safe, manageable, and effective. However, there is a lack of specific guidelines in this localization of IH, about initiation, dosage, and duration of treatment. A longer therapy course for AIH is reported in the literature, compared to their cutaneous counterpart, as described by Elluru et al., with a median duration of 15 months (range 7–34 months). In addition, the age at the end of treatment is debated, thus some authors suggest to continue propranolol at least until 15–18 months of age (12).

We present a case series of 36 patients with airway involvement successfully treated with oral propranolol and discuss our multidisciplinary management.

## Clinical Observations

Demographics and clinical data from 36 patients were collected (Table 1). Twenty-seven out of 36 children (75%) were girls, 22.2% (8/36) had risk factors, including prematurity, twin birth, cesarean section, gestational diabetes, pre-eclampsia, and intrauterine fetal distress. Cutaneous distribution included beard area, but also tragus, sternum, helix, tongue, parotid, nose, and genitalia. A total of 36.1% of patients (13/36) had no cutaneous involvement. In our cases series, only two children were affected with PHACES syndrome. Respiratory symptoms were absent in 13.9% of patients (5/36). Age at onset of cutaneous and/or respiratory symptoms ranged from birth to 90 days of age (average 30.9 and median 30). Therapy was started from 1 to 10 months of age (average 3.5 and median 3) with a total duration ranging from 6 to 26 months (average 15.36 and median 15) and suspension at months 10–54 of age (average 20 and median 19). Additional therapy with steroids was employed in the 27.8% of patients (10/36). Twenty-eight of the 36 patients were brought to a maintenance dose of 2 mg/kg/d, whereas seven patients required 3 mg/kg/d and 1 with PHACES syndrome had to maintain a dosage of 1 mg/kg/d. Five patients (13.9%) had relapses at suspension and restarted oral therapy. Endoscopy characteristics were also collected (Table 2). In our series all patients with recurrence of symptoms after the end of the therapy had endoscopic evidence of persistence of the IH, with subglottic obstruction ≥30% (range 30–50%). The mean percentage of airway obstruction before treatment was 50.29% (range 10–80%) and the mean percentage after treatment was 7% (range 0–30%). Furthermore, subglottic IHs had mostly an eccentric localization pattern (64.7% of cases). In 10 patients we found extra-laryngeal airway localizations of AIHs: pharynx in seven cases and trachea in three patients.

**Table 1:** Demographics and clinical data from 36 patients.

**Case**	**Sex**	**Risk factors**	**Signs**	**Symptoms**	**Onset age**	**Therapy start age**	**Dosage**	**Therapy end age**	**Length of therapy**	**Relapse**	**Other therapy**	**Associated syndromes**
1	F	None	Beard distribution, tragus, helix, tongue, and oral mucosa	None	Birth	3 mo.	0,5–3/mg/kg/day	22 mo.	19 mo.	No	None	None
2	F	None	None	Yes	2 mo.	3 mo.	2 mg/kg/day	18 mo.	16 mo.	Yes, at suspension at 10 mo. of age	Steroids	None
3	M	None	None	Yes	3 mo.	5 mo.	1.5–2 mg/kg/day	22 mo.	17 mo.	No	Steroids	None
4	M	None	Beard, sternum, tongue	Yes	Birth	1 mo.	0.5–3 mg/kg/day	–	–	–	Steroids	None
5	M	Preterm	Beard, helix, sternum	None	7 dd	1 mo.	0.5–1 mg/kg/day	18 mo.	17 mo.	No	Steroids	PHACES
6	F	None	Beard, tragus, left parotids, and thorax	Yes	15 dd	3 mo.	2–3 mg/kg/day	26 mo.	16 mo.	Yes, at suspension at 20 mo.	Steroids	None
7	F	None	Beard tragus, ear, cyrano, tongue	Otorrhea at angioma site	Birth	1 mo.	1–3 mg/kg/day	26 mo.	25 mo.	No	None	None
8	F	None	Right shoulder and axilla	Yes	1 mo.	3 mo.	2–3 mg/kg/day	Still in therapy	Still in therapy	No	None	None
9	M	None	Beard, tragus, lower lip, mucosal	Yes	15 dd	6 mo.	2 mg/kg/day	19 mo.	13 mo.	No	Steroids	None
10	F	None	Segmental unilateral ih, neck, mucosal	Yes	Birth	2 mo.	1–2 mg/kg/day	19 mo.	17 mo.	No	None	None
11	M	Preterm, CS, twin	None	Yes	1 mo.	1 mo.	2 mg/kg/day	20 mo.	18 mo.	Yes, at suspension at 13 mo. of age	Steroids	None
12	F	None	None	Yes	1 mo.	7 mo.	1.5–2 mg/kg/day	24 mo.	17 mo.	No	Steroids	None
13	F	Preterm	Temporal and parietal bilateral, cyrano, lower lip, oral mucosa, neck	None	3 mo.	3 mo.	0.5–2 mg/kg/day	26 mo.	23 mo.	No	No	PHACES, hepatic and cerebral hemangiomas
14	F	None	Segmental unilateral, preauricular, neck	None	Birth	8 mo.	2 mg/kg/day	15 mo.	8 mo.	No	Oral steroid and il, embolization	None
15	M	None	Preauricular, ear, beard, oral mucosa	Yes	1 mo.	4 mo.	2 mg/kg/day	54 mo.	26 mo.	Yes, at suspension at 20 mo. and 48 mo. of age	Steroids	None
16	F	None	Beard, tragus	Yes	Birth	3 mo.	2 mg/kg/day	13 mo.	10 mo.	No	Steroids	None
17	F	Preterm	Segmental unilateral right eye, temporal, parietal, cyrano, conjunctival	Yes	1 mo.	3 mo.	1–3 mg/kg/day	25 mo.	22 mo.	Yes, at suspension at 19 mo. of age	Steroids	None
18	F	None	Preauricular bilateral, beard	Yes	2 mo.	2 mo.	2 mg/Kg/day	10 mo.	8 mo.	None	Steroids	None
19	F	None	None	Yes	2 mo.	4 mo.	2–3 mg/kg/day	19 mo.	15 mo.	None	Steroids	None
20	F	CS	Beard, sacral, right leg	None	birth	2 mo.	2 mg/Kg/day	15 mo.	13 mo.	None	None	None
21	F	None	Genital	Yes	2 mo.	3 mo.	2 mg/kg/day	18 mo.	15 mo.	None	Steroids	None
22	F	CS	Segmental parietal, temporal, eye, shoulder left	Yes	1 mo.	1 mo.	1–2 mg/kg/day	22 mo.	21 mo.	None	None	None
23	F	None	Tongue, cheek, preauricular, lower lip	Yes	Birth	1 mo.	0.5–2 mg/kg/day	18 mo.	17 mo.	None	None	None
24	F	None	None	Yes	1 mo.	10 mo.	2 mg/Kg/day	24 mo.	14 mo.	None	None	None
25	F	Preterm, gestosis	None	Yes	Birth	3 mo.	2 mg/kg/day	18 mo.	15 mo.	None	Steroids	None
26	F	None	Beard, tragus tongue, neck	Yes	Birth	2 mo.	0.5–2 mg/kg/day	18 mo.	16 mo.	None	Steroids	None
27	F	None	None	Yes	2 mo.	4 mo.	2 mg/Kg/day	28 mo.	14 mo.	None	Steroids	None
28	F	None	Beard, preauricular right, lower lip, mucosal	Yes	1 mo.	2 mo.	2 mg/kg/day	14 mo.	12 mo.	None	Steroids	None
29	M	None	None	Yes	3 mo.	5 mo.	2 mg/kg/day	23 mo.	18 mo.	None	Steroids	None
30	F	None	None	Yes	1 mo.	4 mo.	2 mg/kg/day	10 mo.	6 mo.	None	None	None
31	F	None	Beard, lower lip, tragus right	Yes	2 mo.	2 mo.	2 mg/kg/day	25 mo.	23 mo.	None	Steroids	None
32	F	None	None	Yes	2 mo.	4 mo.	2 mg/kg/day	17 mo.	13 mo.	None	Steroids	None
33	M		None	Yes	–	6 mo.	2 mg/kg/day	20 mo.	14 mo.	None	Steroids	None
34	F	None	Abdomen and foot	Yes	1 mo.	5 mo.	2 mg/kg/day	20 mo.	15 mo.	None	Steroids	None
35	M	Preterm	None	Yes	2 mo.	7 mo.	2 mg/kg/day	20 mo.	13 mo.	None	Steroids	None
36	F	None	Beard, sternum, lower lip	Yes	2 mo.	3 mo.	2 mg/kg/day	17 mo.	14 mo.	None	Steroids	None

*CS, Cesarean section; d, days; M, male; F, female; mo, months*.

**Table 2:** Endoscopic characteristics of the 36 patients.

**Case**	**% of obstruction before therapy**	**Pattern of obstruction**	**N**°** of airway endoscopies**	**% of obstruction after therapy**	**Other airway's localization**
1	20%	Eccentric	3	0%	Trachea
2	50%	Eccentric	3	20%	None
3	50%	Eccentric	5	10%	None
4	80%	Concentric	4	0%	Pharynx
5	40%	Eccentric	2	0%	Pharynx
6	40%	Concentric	4	0%	None
7	–	–	0	–	None
8	80%	Concentric	3	–	None
9	50%	Eccentric	3	10%	Pharynx
10	40%	Eccentric	2	20%	Pharynx
11	70%	Eccentric	7	0%	None
12	60%	Concentric	3	0%	None
13	50%	Eccentric	3	10%	None
14	–	–	0	–	None
15	60%	Concentric	5	0%	None
16	50%	Eccentric	3	0%	None
17	50%	Concentric	4	10%	None
18	10%	Eccentric	1	–	Pharynx
19	60%	Concentric	2	10%	None
20	10%	Eccentric	1	–	None
21	70%	Eccentric	2	20%	None
22	50%	Eccentric	3	20%	None
23	20%	Eccentric	2	0%	None
24	70%	Concentric	2	10%	None
25	60%	Eccentric	2	0%	Trachea
26	30%	Concentric	2	10%	Pharynx
27	70%	Concentric	2	10%	None
28	30%	Eccentric	3	0%	Pharynx
29	50%	Eccentric	5	10%	None
30	40%	Eccentric	3	0%	None
31	40%	Concentric	4	0%	Trachea
32	70%	Eccentric	5	0%	None
33	60%	Eccentric	3	30%	None
34	40%	Eccentric	5	20%	None
35	70%	Eccentric	3	0%	None
36	70%	Concentric	1	–	None

*M, male; F, female*.

## Discussion

Airway IHs cause functional damage due to upper airway obstruction leading to respiratory insufficiency and life-threatening complications in the absence of treatment. They are frequently associated with a cutaneous distribution in a known fashion (3). Although, as seen in our case series and in the literature, they can present without cutaneous involvement. There is a lack of guidelines and consensus on diagnosis and treatment that often depend on local experience of dermatologists, ear-nose-throat specialists (ENT), and broncopneumologists. Propranolol is the mainstay of treatment; however, it is still preceded by oral steroid courses especially in non-reference centers. B-blockers are rapidly efficacious, thus, oral steroids treatment should not be administered or discontinued as soon as propranolol is started. A prompt diagnosis and an early specific treatment are crucial to prevent hospitalization with repeated laryngoscopy, intubations, and prolonged steroid treatment, with known side effects and delay on vaccination calendar. Other imaging modalities such as magnetic resonance imaging (MRI) or computerized tomography (CT) should be reserved for cases with suspected PHACES syndrome, or to investigate differential diagnoses. In some cases, our patients underwent radiological evaluation by MRI, with very accurate results similar to the endoscopic findings for the head and neck IH, except for the laryngeal manifestations, in particular the subglottic ones, for which the airway endoscopy is essential, both in confirming the diagnosis and the obstruction percentage (7, 11).

AIHs must be considered in cases of unexplained inspiratory stridor and/or dyspnea unresponsive to traditional treatment and recurrence. Stridor is a high-pitched sound, caused by total or subtotal upper airway obstruction. Biphasic stridor heard during both phases of respiration is typical of subglottic diseases. The subglottis includes the cricoid cartilage, which is prone to complications, being rigid and fixed. Differential diagnosis of subglottic obstruction includes inflammatory croup, reflux, subglottic obstruction, intubation granuloma, cysts, congenital cricoid malformation, foreign body, and infantile hemangioma (13). The specific timing of presentation of the IHs is very indicative and the endoscopic evaluation is crucial. Patients could be misdiagnosed as presentation is similar and other conditions improve with steroid therapy, both by inhalation and systemic delivery. Therefore, recurrence and worsening of respiratory symptoms despite therapies are suspicious for IH.

In our retrospective study, we present data about the management of AIHs in 36 patients successfully treated with oral propranolol (Table 1). The median length of treatment was 15 months, which is consistent with data in the literature (12, 14). In our patients, therapy was discontinued at months 10 to 54 of age (average 20 and median 19), in keeping with previous case series (12). Based on our experience, we suggest starting oral propranolol as soon as possible, even before 5 weeks of age (15), and to stop it at least at 18 months of age, if started promptly or after 15 months of duration in cases in which the treatment is started after 3 months of age. Treatment efficacy and safety monitoring could be based on symptoms and vital parameters, reserving endoscopy only for diagnosis and at the end of treatment.

Some authors suggest dose-tapering of oral propranolol, while recent guidelines and also our personal experience confirm that it should be interrupted abruptly (1). Elluru et al. reported that six patients required additional medical or surgical approach for persistent respiratory symptoms, but none of our patients presented stridor after the first 1–2 weeks of treatment. In our case series, additional steroids therapy was employed in 10 patients, mainly in severe cases, while awaiting the initiation of propranolol therapy and discontinued afterward. In addition, we did not have propranolol non-responder in our cohort (14). However, different authors reported variable percentages of failure-cases, ranging from 9 to 22% (12, 14, 16). In our opinion these percentages are over-represented, might be due to delay of diagnosis and therapy start, duration of treatment, and age at discontinuation (17, 18).

Relapses occurred in five patients (13.9%), due to (i) delay of treatment onset, (ii) wide extension of the cutaneous IH, (iii) association with deep component of the skin lesion, (iv) involvement of the parotid gland, and (v) reduced duration of treatment. Interestingly, in three out of these five children, oral propranolol was administered at the dosage of 3 mg/kg/day. Moreover, AIH recurred only in two out of 28 patients on 2 mg/kg/day regimen, thus in our opinion, relapses might not be due to dosage. Percentage of relapses in our population is consistent with literature data (12). Causes of relapses are still debated, indeed recurrences in our patients frequently occurred both in early therapy commencement (4/5) and in longer duration (3/5). For this reason, a clinical follow-up after treatment discontinuation is mandatory.

Only two out of 10 patients with large segmental IH of the face were diagnosed with PHACES syndrome compared to a higher percentage in literature data (47%) (4, 6). When PHACES syndrome is suspected, it is necessary to perform a full workup, including cardiologic, ophthalmologic, and neurologic investigation with appropriate imaging (19). Meanwhile, in the case of suspected PHACES with severe airway obstruction, treatment should be started at the lowest dose, divided in some cases in three administration, in order to prevent stroke occurrence (20), then adjusted based on the finding of MRI on cerebro-vascular anomalies (21). In our patients, the same plan was adopted for severe preterm babies. Several protocols for the management of AIHs have been proposed in the literature, but none has been standardized thus far (14, 22, 23). Over the years, our experience in the diagnostic, treatment, and follow-up indications has improved. When propranolol therapy was first introduced, our approach was more invasive, with several and close endoscopic procedures during treatment. We also observed that the trend of this pathology is systematic and therefore we believe that endoscopy does not have to be repeated during treatment, but it should be performed 1 month after discontinuation of propranolol to evaluate the results and possible recurrence of AIH, avoiding general anesthesia and controlling public health-care costs. Of course, further endoscopic evaluation should be carried out in case of relapse of respiratory symptoms and therefore persistence of AIH, with the need to continue the pharmacological treatment. In our experience, finding of residual disease with obstruction of the respiratory space up to 20% does not contraindicate the end of the therapy.

The limitations of this study are: (i) it is retrospective, (ii) a reduced number of cases, and (iii) unexplained relapses causes in a limited number of patients.

In conclusion, the diagnosis of AIH should be suspected in cases of biphasic stridor and/or typical cutaneous involvement, and always confirmed with airway endoscopy. Oral propranolol is the mainstay of therapy and should not be preceded by oral steroid, due to its rapid efficacy. Treatment should be started as soon as possible with a duration for at least 15 months and/or reaching 18 months of patient age. Clinical follow-up after discontinuation of therapy is mandatory to promptly manage possible relapses.

## Data Availability Statement

The original contributions presented in the study are included in the article/supplementary material, further inquiries can be directed to the corresponding author.

## Author Contributions

All authors listed have made a substantial, direct, and intellectual contribution to the work and approved it for publication.

## Conflict of Interest

Two of the authors ME and AD of this publication are members of the European Reference Network ERN-SKIN and of Vascular Anomalies Working Group VASCA WG of the ERN for rare Multisystemic Vascular Diseases VASCERN. The remaining authors declare that the research was conducted in the absence of any commercial or financial relationships that could be construed as a potential conflict of interest.

## Publisher's Note

All claims expressed in this article are solely those of the authors and do not necessarily represent those of their affiliated organizations, or those of the publisher, the editors and the reviewers. Any product that may be evaluated in this article, or claim that may be made by its manufacturer, is not guaranteed or endorsed by the publisher.
